# Effectiveness of different fiber post removal techniques and their influence on dentinal microcrack formation

**DOI:** 10.1007/s00784-021-04338-0

**Published:** 2021-12-10

**Authors:** Franziska Haupt, Insa Riggers, Frank Konietschke, Tina Rödig

**Affiliations:** 1 Department for Preventive Dentistry, Periodontology and Cariology, University Medical Center Göttingen, Robert-Koch-Str. 40, 37075 Göttingen, Germany; 2 Charité - University Medical Center Berlin, Corporate Member of Freie Universität Berlin, Humboldt-Universität Berlin, and Berlin Institute of Health, Institute of Biometry and Clinical Epidemiology, Berlin, Germany; 3 Berlin Institute of Health (BIH), Berlin, Germany

**Keywords:** Dentin loss, Effectiveness, Fiber post removal, Induction of microcracks, Micro-computed tomography

## Abstract

**Objectives:**

The aim of this study was to evaluate the effectiveness of different fiber post removal techniques and to correlate dentinal loss with microcrack formation.

**Materials and methods:**

Forty-five extracted single-rooted teeth were root canal treated and fiber posts were adhesively luted. Specimens were divided into three groups (*n* = 15) according to the removal technique: long-shaft round bur (EndoTracer #08, Komet, Lemgo, Germany), SonicFlex Endo (KaVo, Biberach, Germany), DT Post Removal Kit (VDW, Munich, Germany). Roots were scanned before post cementation and after post removal using micro-computed tomography. Dentin loss, residual luting material, working time, and the induction of microcracks were assessed. Statistical analysis was performed by using multiple contrast tests (max-*t* tests, *α* = 0.05). Correlations between parameters dentin loss/new microcracks and dentin loss/residual material were calculated using Kendall’s tau.

**Results:**

Post removal with SonicFlex Endo resulted in the highest amount of removed dentin with significant differences to the round bur and the DT Post Removal Kit. No technique was found to completely remove the post and luting material. All techniques induced microcracks with the DT Post Removal Kit presenting the highest number of new defects. No correlation between dentin loss and new microcracks was observed. Deviations from the original root canal occurred in all groups, but no perforation was observed.

**Conclusions:**

All techniques resulted in dentin loss, residual luting material, and the formation of microcracks. However, no correlation between dentin loss and the induction of microcracks was observed.

**Clinical relevance:**

As all techniques resulted in microcrack formation and dentin loss, this study emphasizes the risk of iatrogenic damage due to post removal procedures.

## Introduction

During the last decades, fiber posts in combination with composite cores have gained more popularity for reconstruction of teeth with extensive hard tissue loss [1, 2]. Reasons for their popularity are the mechanical and esthetic characteristics as well as the more favorable mode of failure compared to metal posts [3–5]. In case of retreatment, posts should be safely removable with a low risk of iatrogenic errors, such as deviations and perforations. Additionally, it is still a matter of debate, whether root canal procedures may induce the formation of dentinal microcracks assumed as precursors of vertical root fractures, which inevitably result in tooth extraction [6, 7]. In recent literature, the influence of root canal preparation on the formation and propagation of dentinal defects is repeatedly and controversially discussed [8–14]. Furthermore, it is widely accepted that fracture susceptibility of teeth increases with extensive loss of dentin [7, 15–17]. Nevertheless, despite the more aggressive behavior of post removal drills compared to shaping instruments, there is a lack of data available regarding microcrack induction due to post removal procedures [11, 18]. Previous studies have investigated the effectiveness of post removal techniques considering the amount of dentin loss and residual luting material [19–21]. However, there is an absence of evidence regarding the interrelations between tooth substance loss due to different removal techniques and formation of dentinal defects.

Therefore, the first aim of this study was to evaluate the induction of dentinal microcracks due to post removal procedures. Secondly, the effectiveness of three different removal techniques in terms of dentin removal, residual luting material, and working time was assessed. Two null hypotheses were formulated: All post removal techniques perform equally regarding their effectiveness and induction of dentinal defects. There are no correlations between the following parameters: dentin loss/new microcracks and dentin loss/residual luting material.

## Material and methods

### Sample size calculation

Based on data of a previous published study [19], sample size calculation was carried out using G*Power 3.1.9.7 software (Heinrich-Heine-Universität, Düsseldorf, Germany) with *α* = 0.05, a power of 0.95, and an effect size *d* of 1.40 resulting in a required size of 12 samples per group.

### Specimen selection, preparation, and post insertion

After approval of the local ethics committee (No. 27/8/13), forty-five human maxillary incisors, extracted for periodontal reasons, were included in the present study and stored in tap water until usage. Criteria for inclusion were as follows: single-rooted teeth with straight roots, no previous root canal treatment, complete root development, and at least 15 mm root length. Access to the root canal system was performed and root length was determined using a dental operating microscope (10 × magnification; Zeiss pico, Zeiss, Jena, Germany) by inserting a size #15 K-File into the root canal until the tip of the instrument was just visible at the apical foramen. Teeth were decoronated with a diamond bur to a standardized root length of 15 mm, resulting in a working length of 14 mm. Root canals were prepared with reciprocating NiTi instruments (Reciproc 50, VDW, Munich, Germany) according to the manufacturer’s instructions. After 3 pecking motions, 2 mL NaOCl (3%) was used as irrigant. After a final flush of 5 mL citric acid (10%) and 5 mL NaOCl (3%), root canals were dried with paper points and the apical 4 mm of all canals was obturated using the corresponding gutta-percha and sealer (2Seal, VDW) with warm vertical compaction technique. If necessary, root canals were prepared in the coronal region with Gates-Glidden burs size 3 and irrigated with 2 mL NaOCl (3%) until fitting of the fiber post (X·Post size 1, Dentsply Sirona, Baillaigues, Switzerland) was established. Teeth were stored in 100% humidity at 37 °C for 1 week to allow setting of the sealer. Until scanning procedures, specimens were kept in water for at least 24 h to ensure moisture penetration into dentin. Prior to micro-computed tomography (micro-CT) scans, specimens were dried at room temperature and 27% humidity for 2 h to allow detection of microcracks [22]. Specimens were placed in a sample holder which was tightly closed with plastic foil to keep the humidity constant during scan duration. Roots were scanned using a micro-CT scanner (SkyScan 1272; Bruker-microCT, Kontich, Belgium) at 80 kV and 125 µA with an isotropic resolution of 5 µm with a 180° rotation around the vertical axis with a rotation step of 0.15°, a camera exposure time of 3518 ms and frame averaging of 3. X-rays were filtered with a 1-mm-thick aluminum filter. Images were reconstructed using NRecon v.1.7.5.9 software (SkyScan 1272; Bruker-microCT) with 40% beam hardening correction, ring artefact correction of 18, and postalignment between − 1 and + 5, resulting in the acquisition of 1756–2089 transverse cross-sections per tooth. Prior to post insertion, all root canals were cleaned with ethyl alcohol, conditioned with 36% phosphoric acid for 15 s, rinsed with distilled water for 20 s, and dried with paper points. All posts were shortened to a length of 10 mm using a water-cooled diamond bur and a mixture of Prime&Bond XP (Dentsply Sirona) and Self-cure Activator (1:1) was applied for 20 s to the root canal wall and posts. Subsequently, root canals were filled with Core-X flow (Dentsply Sirona) and posts were placed immediately to full depth of 10 mm. Adhesive seal of the coronal end of the post was ensured and light polymerization was applied for 40 s. Specimens were then stored again in 100% humidity at 37 °C for at least 1 week.

Preoperative root canal volume and surface area were estimated from the binarized images by using CTan v.1.20.3.0 software (Bruker-microCT). Based on the assessed parameters (root canal volume and surface area), specimens were assigned to three homogeneous groups (*n* = 15) according to the post removal technique:

Long-shaft round bur (EndoTracer size 08, Komet, Lemgo, Germany), 8000 rpm.

SonicFlex Endo, diamond-coated conical tip #67 (KaVo, Biberach, Germany), 3.5 bar, water-cooled.

DT Post Removal Kit (VDW), pilot drill and carbide drill at 2000 rpm.

Homogeneity of the groups regarding preoperative volume and surface area was confirmed using one-way analysis of variance (P_volume_ = 0.96, P_surface_ = 0.99).

### Post removal

Each root was wrapped with a single layer of textile adhesive tape (0.3 mm; PERFEKT, tesa, Norderstedt, Germany) and embedded in acrylic resin (Technovit 4071, Heraeus, Hanau, Germany) set in an acrylic tube. The root was then removed and the tape was peeled off. Polyether impression material (Impregum, 3 M ESPE, Seefeld, Germany) was placed in the resin, the tooth was reinserted, and the excess material was removed with a scalpel blade. So, the impression material replaced the space created by the tape and represented a simulated periodontal ligament [23].

Post removal procedures were carried out using a dental operating microscope (10 × magnification; Zeiss pico, Zeiss) by one single trained operator. All instruments were moved in axial direction with slight pressure to a length of 10 mm. Time elapsed during post removal was recorded using a stopwatch. Severe failure during removal procedures (perforation, deviation) was recorded.

### Micro-CT analysis

**Fig. 1:**
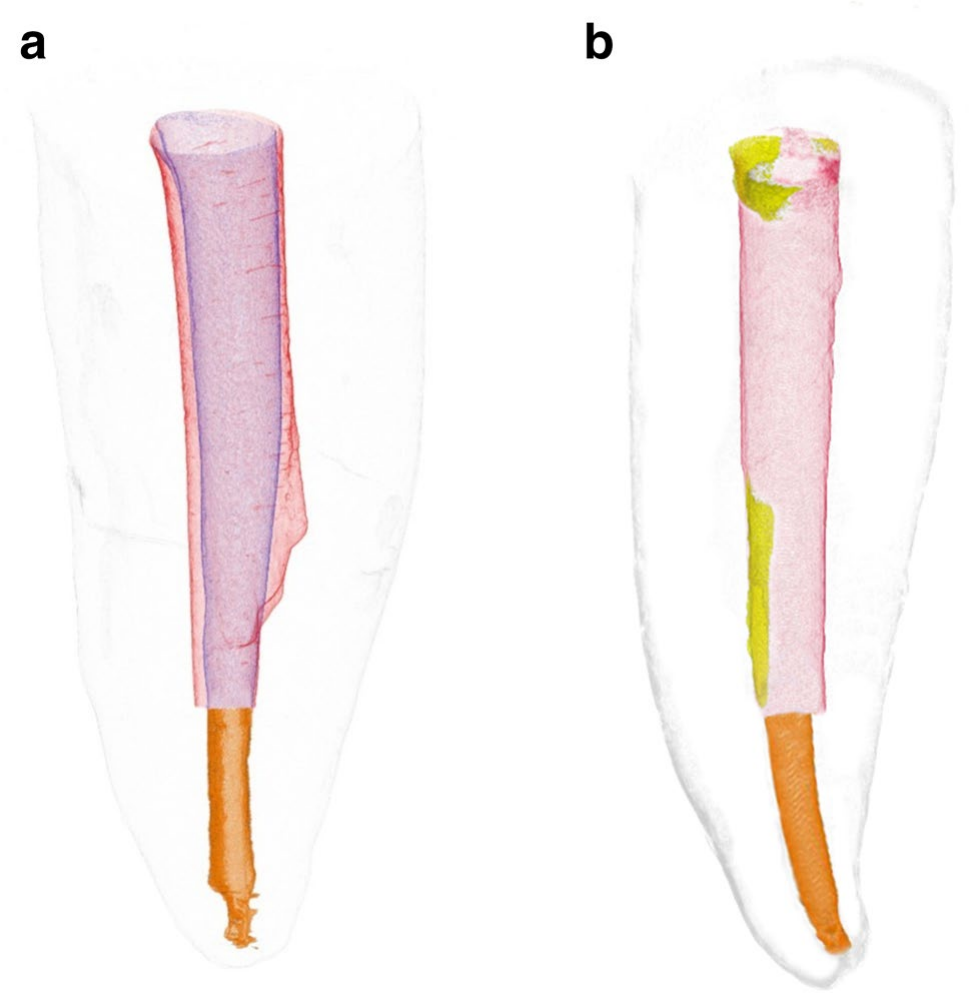
**a** Representative three-dimensional reconstruction of the apical gutta-percha (orange) and root canal volumes before (violet) and after (red) post removal with the DT Post Removal Kit indicating dentin loss. **b** Representative three-dimensional reconstruction of the apical gutta-percha (orange), postoperative root canal volume (red), and residual luting material (yellow) after removal with the DT Post Removal Kit

Specimens were again dried at room temperature for 2 h and then submitted to a postoperative scan and reconstruction applying the initial parameter settings. Reconstructed data sets before and after post removal procedures were co-registered in DataViewer v.1.5.4.0 software (Bruker-microCT) using a pseudo-3D registration tool. CTan v.1.20.3.0 software (Bruker-microCT) was applied to calculate quantitative parameters. Removal of dentin and volumes of residual material were assessed by subtracting pre- and postoperative values (Fig. 1a, b).

### Dentinal microcrack evaluation

Aiming to identify the presence of dentinal defects, all postoperative cross-sectional images between the coronal end of the root and the filling material (*n* = 86,444) were screened by two previously calibrated examiners who were blinded to the experimental groups. In case of microcrack observation, the corresponding preoperative cross-sectional image was inspected to verify the pre-existence of the defect [12]. If there was disagreement between the observers, a consensus was achieved by re-evaluation of the images together [10]. Compared to the preoperative image, defects were distinguished between pre-existing defects and new defects. According to the location, new microcracks were classified into defects initiating from the external surface, from the internal surface and continuous defects.

### Statistical analysis

Statistical analysis was performed by using parametric and nonparametric multiple contrast tests (max-*t* tests). The level of significance was set at *α* = 0.05, where *P*-values were adjusted for pairwise comparisons. Correlation coefficients between parameters dentin loss/new microcracks and dentin loss/residual material were calculated using Kendall’s tau.

## Results

**Fig. 2:**
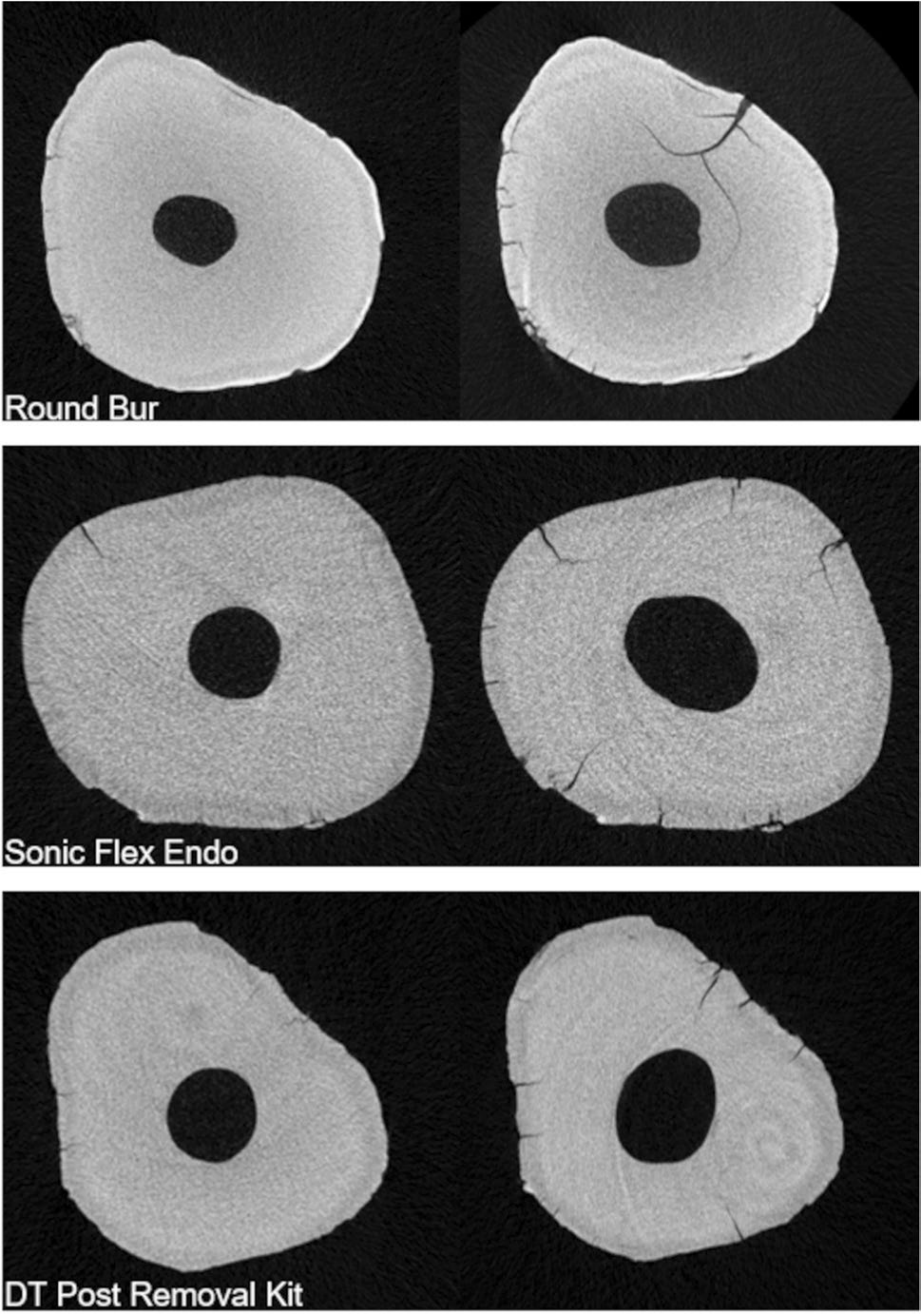
Representative pre- and postoperative cross-sectional images showing the presence of new microcracks after post removal

The results of 3D analysis are detailed in Table 1. Comparison between post removal techniques revealed statistically significant differences regarding all tested parameters (*p* < 0.05). With respect to the experimental group, mean percentages of preoperative slices with microcracks were 33.07 ± 29.14 (round bur), 50.65 ± 4.51 (SonicFlex Endo), and 60.94 ± 41.21 (DT Post Removal Kit). Concerning defect location, all pre-existing defects initiated from the external root surface. Mean percentages of slices with new microcracks as well as the local distribution are shown in Table 1. Representative cross-sectional images before and after post removal are illustrated in Fig. 2. Regarding working safety, no perforation was observed. Deviations from the original root canal occurred in all groups (round bur: 2 × , SonicFlex Endo: 1 × , DT Post Removal Kit: 2x). Correlation analysis between parameters dentin loss/new microcracks and dentin loss/residual material provided slight negative correlations (Kendall’s tau coefficients: τ_DentinLoss/Microcracks_ =  − 0.17, *P* = 0.11; τ_DentinLoss/ResidualMaterial_ =  − 0.34, *P* = 0.001).

**Table 1:** Means ± standard deviation and *medians* of percentages of slices with new defects, morphometric data after post removal procedures and removal time for the three experimental groups. Letters indicate statistically significant differences among groups (*P* < 0.05)

		Round Bur	Sonic Flex Endo	DT Post Removal Kit
% slices with new microcracks		49.07 ± 29.39^a,b^ *46.88*	39.74 ± 27.01^b^ *34.69*	64.63 ± 23.49^a^ *64.95*
% Distribution of new microcracks according to location	Outer surface	75.29 ± 36.51^a^ *100*	86.67 ± 35.19^a^ *100*	98.77 ± 4.76^a^ *100*
	Inner surface	0.80 ± 3.08^a^ *0.00*	0.00 ± 0.00^a^ *0.00*	0.00 ± 0.00^a^ *0.00*
	Continuous	17.24 ± 28.57^a^ *0.00*	0.00 ± 0.00^a^ *0.00*	1.23 ± 4.76^a^ *0.00*
Dentin loss (mm^3^)		5.61 ± 1.74^a^ *5.56*	9.56 ± 3.02^b^ *9.93*	5.70 ± 2.72^a^ *4.79*
Residual luting material (mm^3^)		0.06 ± 0.06^a,b^ *0.04*	0.06 ± 0.01^a^ *0.02*	0.20 ± 0.21^b^ *0.13*
Removal time (min)		12.65 ± 4.75^a^ *11.97*	8.86 ± 2.51^b^ *8.67*	7.63 ± 0.77^b^ *7.52*

## Discussion

In case of orthograde retreatment, a post should be safely removable with a low risk of iatrogenic errors [24, 25]. In recent times, there have been several studies evaluating effectiveness, working time, and risks of fiber post removal [21, 24–27], but only few studies have additionally assessed the amount of removed dentin [18–20, 28]. However, results of the latter are difficult to compare as they highly differentiate from each other regarding the applied methodology. As in previous studies, micro-CT analysis was used, which allows 3D reconstruction of teeth and precise calculation of substance loss due to endodontic procedures [18, 20]. In the present study, glass fiber posts were removed with three different removal systems. As these posts are adhesively luted to root dentin by forming a monoblock [29], post removal may be accomplished by disintegrating the post along the fiber bundles embedded in an epoxy-resin matrix. Therefore, special post removal kits are available, which usually consist of a pilot drill to create an initial access followed by a carbide bur to drill out the entire post. Compared to previous studies, the present study confirms the high efficiency of the DT Post Removal Kit with preservation of sound dentin [18, 19]. Nevertheless, removal with the DT Post Removal Kit resulted in significantly more residual material compared to the use of a diamond-coated sonic tip. Regardless of the removal technique, the amount of residual luting material correlated significantly negative with the extent of removed dentin. The correlation is clearly demonstrated as the use of the sonic tip resulted in the highest amount of removed dentin. One reason might be the conical geometry with a coronal diameter of 1.5 mm, compared to diameters of 0.8 mm and 1.3 mm for the round bur and the DT Post Removal Kit, respectively. Depending on the removal technique, previous studies reported mean volumes of residual material between 1.1 ± 6.8 and 3.3 ± 4.0 mm^3^ [18, 19], which appears to be remarkably more than the values measured in the present study. However, in the present study, post removal was carried out under tenfold magnification using a dental operating microscope, which might be an explanation for these differences. Nevertheless, decoronation of extracted teeth presents a limitation of this in vitro setting as post removal is facilitated compared to clinical situations, in which retreatment is predominantly performed in teeth with full-coverage restorations and limited access. Whether these minimal volumes of residual material are realistic in a clinical setting or have an impact on clinical outcomes, remains unclear.

Apart from reduction of the microbial load, preservation of hard tissue should be the ultimate goal during endodontic procedures. Especially post removal implies the risk of iatrogenic errors, such as deviations from the original root canal and perforations [19], thus decreasing the long-term survival of the retreated tooth. Additionally, excessive removal of cervical dentin (danger zone) increases the risk of substantial damage which detrimentally affects tooth fracture resistance [30]. Furthermore, dentin thickness of less than 1.3 mm is associated with a greater likelihood of vertical root fracture of endodontically treated teeth [31]. In recent literature, cone beam computed tomography and 3D-printed guiding templates have enabled the location of calcified canals, facilitating endodontic treatment of those teeth and preserving hard tissue during access cavity preparation [32]. Equally, *guided endodontics* may be applied during endodontic retreatment as an efficient method to access a root canal through a fiber post [33]. Nevertheless, further studies are needed to validate clinical benefits.

In recent literature, several studies have evaluated the influence of endodontic procedures on formation and propagation of dentinal defects [9, 11–14, 34]. Basically, there are two different methodologies for microcrack evaluation: destructive tests are carried out by postoperative sectioning of the specimens at certain intervals. These slices are submitted to stereoscopic analysis regarding the absence or presence of microcracks [9, 11, 34]. In contrast, non-destructive micro-computed tomography allows assessment of pre- and postoperative scans at high resolutions and hundreds of slices per root can be observed resulting in higher accuracy and more reliable data [13]. Furthermore, specimens serve as their own control with no need of root sectioning, which might have deleterious effects on root dentin [34]. Unfortunately, the use of the different methodologies among the literature results in certain inhomogeneity and poor comparability. In the present study, micro-CT scans were performed at a high resolution of 5 µm and a rotation step of 0.15°. On the one hand, these parameters allow a detailed examination of the roots regarding microcrack formation; on the other hand, there is a risk of dehydration of dentin due to the long scan duration of 3.5 h.

Apart from being induced by root canal procedures, microcrack formation can occur due to dehydration of dentin, which may produce stresses exceeding the strength of dentin [35–37]. In a previous study, it was shown that dentin slices of 3 mm thickness may present dehydration-induced microcracks within 5 min after sectioning. However, other slices did not exhibit crack formation even within 24 h dry time. In the same study, micro-CT analysis of entire roots revealed microcracks in 60% of the specimens after 7 days of dehydration [36]. However, within 1 week, it is difficult to determine the point in time at which microcrack formation occurred. In contrast, a present study evaluating the influence of moisture content of extracted teeth on the detection of microcracks using micro-CT imaging demonstrated that microcracks were blacked out due to excessive moisture condition of dentin, leading to false-negative results [22]. In fact, drying of extracted roots for 2 h enables microcrack detection and dehydration for up to 24 h did not induce new microcracks [22]. Therefore, in the present study, roots were dried at room temperature for 2 h to facilitate microcrack detection. During the high-resolution scan, the sample holder was tightly closed with plastic foil to prevent further dehydration of specimens. Nevertheless, it is still challenging to maintain a balance between the visibility of microcracks and the formation of new defects due to dehydration.

It is well known that the periodontal ligament is able to act as an intermediate cushion element which absorbs applied forces and transfers these into the surrounding bone [38]. In the present study, an artificial periodontal ligament was produced to simulate these functions, but, certainly, it is not able to reflect the natural periodontal apparatus. New approaches for microcrack evaluation included fresh or embalmed cadaveric dento-alveolar bone-blocks, as these models represent the closest to real-life condition by preserving the viscoelastic properties of the periodontal ligament and the surrounding bone, absorbing the forces applied to dental tissues during root canal procedures [39, 40]. However, even if the use of cadaveric models in combination with micro-CT imaging presents the gold standard, sample acquisition is still challenging and might result in small sample sizes and inhomogeneous experimental groups regarding tooth type, root canal volume, or surface area.

Only few studies assessed the effect of post removal techniques on the formation of microcracks with varying results, maybe due to different methodologies, sample size, and instruments for post removal [11, 18]. In the present study, all removal techniques resulted in microcrack formation, with the DT Post Removal Kit showing the highest and the SonicFlex Endo the lowest percent of slices with new defects. The vast majority of new defects initiated from the external surface without significant differences between groups. Correlation analysis between dentin removal and formation of microcracks revealed no significant results. Therefore, it can be assumed that formation of microcracks is not directly related to post removal procedure. Even if dentin removal, especially in the cervical region, increases the fracture susceptibility of endodontically treated teeth [31], formation of microcracks obviously does not depend on substance loss alone. As tooth fracture is a multifactorial occurrence, there are additional reasons apart from dentin removal, leading to microcrack formation and finally resulting in a decreased fracture resistance. The influences of masticatory forces and parafunctions, tooth position, adjacent teeth, patient’s age, and different coronal restorations were not examined in the present study. One reason for the formation of new dentinal defects might be the procedure itself by applying forces in coronal-apical directions during post removal. However, further studies are needed to investigate stress levels from applied loads during post removal as well as the influence of dentin removal on microcrack formation under realistic clinical conditions.

## Conclusion

Within the limitations of the present study, all post removal techniques resulted in dentin removal with the sonic tip being the most invasive method. No technique was found to completely remove the post and luting material. All techniques resulted in formation of microcracks mainly on the root surface but no correlation with the amount of removed dentin was observed. Further studies are needed to evaluate the impact of post removal procedures on dentin under realistic clinical conditions.
